# Simulated seal scarer sounds scare porpoises, but not seals: species-specific responses to 12 kHz deterrence sounds

**DOI:** 10.1098/rsos.170286

**Published:** 2017-07-19

**Authors:** Lonnie Mikkelsen, Line Hermannsen, Kristian Beedholm, Peter Teglberg Madsen, Jakob Tougaard

**Affiliations:** 1 Department of Bioscience, Aarhus University, Roskilde, Denmark; 2 Zoophysiology, Department of Bioscience, Aarhus University, Aarhus, Denmark

**Keywords:** acoustic harassment devices, seal scarer, harbour porpoise, harbour seal, mitigation, pile driving

## Abstract

Acoustic harassment devices (AHD) or ‘seal scarers’ are used extensively, not only to deter seals from fisheries, but also as mitigation tools to deter marine mammals from potentially harmful sound sources, such as offshore pile driving. To test the effectiveness of AHDs, we conducted two studies with similar experimental set-ups on two key species: harbour porpoises and harbour seals. We exposed animals to 500 ms tone bursts at 12 kHz simulating that of an AHD (Lofitech), but with reduced output levels (source peak-to-peak level of 165 dB re 1 µPa). Animals were localized with a theodolite before, during and after sound exposures. In total, 12 sound exposures were conducted to porpoises and 13 exposures to seals. Porpoises were found to exhibit avoidance reactions out to ranges of 525 m from the sound source. Contrary to this, seal observations increased during sound exposure within 100 m of the loudspeaker. We thereby demonstrate that porpoises and seals respond very differently to AHD sounds. This has important implications for application of AHDs in multi-species habitats, as sound levels required to deter less sensitive species (seals) can lead to excessive and unwanted large deterrence ranges on more sensitive species (porpoises).

## 1. Introduction

Human activities in offshore areas are on the rise, and underwater noise follows suit with potentially negative consequences for marine life [1]. Powerful anthropogenic sound sources, such as pile driving of wind turbine foundations, have been shown to negatively affect the behaviour of key ecological species such as harbour porpoises (*Phocoena phocoena*) (e.g. [2,3]) and harbour seals (*Phoca vitulina*) [4]; both facing increasing human encroachment in their shallow water habitats. Further, if these animals are close enough to loud transient noise sources, such as pile driving, explosions and airguns, they may also face the risk of temporary or permanent hearing loss (TTS and PTS, respectively) [5,6]. The clear incentive for preventing or reducing such exposures to pile driving and other loud impulsive sound sources has prompted the use of acoustic harassment devices (AHD) or ‘seal scarers’ as mitigation devices. An AHD is accordingly often deployed prior to a pile driving operation to deter animals out to a safe distance before the piling starts. AHDs were, however, originally designed to deter seals from fishing gear and aquaculture installations to avoid depredation on fishes and damage to fishing gear. The effectiveness of these devices for this purpose appears to vary with type of device, characteristics of the emitted sound and context (see review by Götz & Janik [7]). When used to reduce depredation, AHD can potentially inflict large-scale unwanted habitat exclusion of non-target species [7–9], yet, only few studies have addressed the effectiveness of AHDs on different species. Responses to AHDs, quantified as the effective deterrence distance for different species, will probably vary considerably with differences in hearing thresholds among species, as well as differences in ecology, group behaviour and exposure history of individuals.

AHD typically transmit sound in the frequency range 10–40 kHz, well within the range of best hearing in harbour seals [10–12] and harbour porpoises [13,14]. The audiograms of harbour seals and harbour porpoises cross each other approximately around 5 kHz; below 5 kHz, harbour seals have the more sensitive hearing, above 5 kHz, porpoises do. This means that in the frequency range used by many AHDs, harbour porpoises have similar or better hearing than harbour seals. Such differences in hearing thresholds may themselves indicate that porpoises could be expected to react at lower received sound pressure levels and hence at longer ranges than harbour seals. Indeed, a few studies suggest large differences in deterrence distances of AHDs. Harbour seals and grey seals (*Halichoerus grypus*) have been seen to react at ranges from less than 100 m up to about 1 km, depending on characteristics of the device [15–17], whereas porpoises have been shown to react to similar AHDs at ranges of several kilometres [8,9,18,19]. Such differences in response ranges may constitute very large, but unintended impacts on harbour porpoises in fishery-related context where the AHD solely targets seals in order to reduce depredation. In addition to this, for mitigation purposes, far-reaching effects on porpoises beyond the zone where they may be expected to acquire hearing loss may add on top of the primary noise source (pile driving or other) that the ADH serves to protect the animals from. In the worst case, an AHD may thereby have net negative consequences for the individual animals it was intended to protect.

For both applications, it is therefore important to adjust the source parameters of deterrence devices to what is required for an adequate deterrence and protection of both seals and small cetaceans. Unfortunately, the knowledge about diverse responses of different species to the same ADHs is limited (see, however, [20]). Thus, the aim of this study was to quantify and compare the deterrence ranges of harbour porpoises and harbour seals in response to a simulated AHD signal in order to evaluate the effectiveness of AHDs as a deterrence device for these two species. Both species have been well studied with respect to general hearing physiology and are the two principally relevant species in assessments of impact from pile driving and other loud impulsive sound sources in northern European waters. We show here that the two species respond very differently to playback of a 12 kHz simulated AHD signal.

## 2. Material and methods

**Figure 1.**
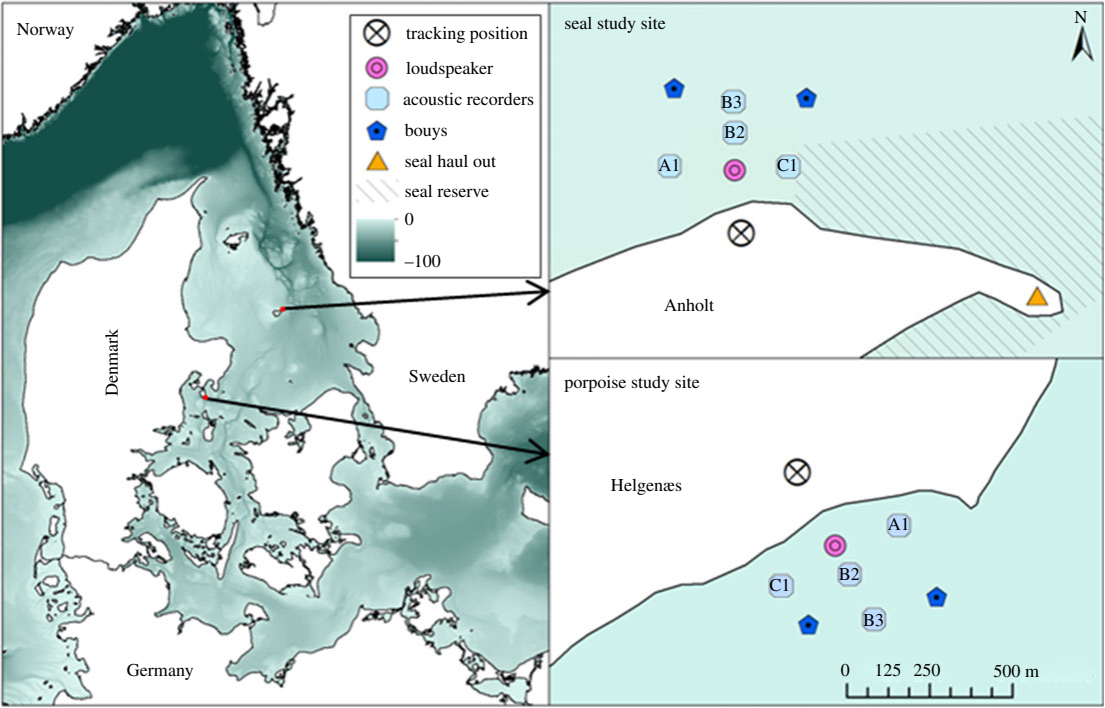
The two study sites. (Top) The northeastern tip of Anholt (seals). (Bottom) The southern coast of Helgenæs (porpoises). Station names of the acoustic recorders are indicated.

Two studies were conducted with similar set-ups to investigate responses of harbour porpoises and harbour seals to simulated AHD sounds. The study on harbour porpoises was conducted at Helgenæs, Denmark (figure 1), 29 June–18 July 2015, where the high point ‘Burs klint’ (56°6.153′ N, 10°32.23′ E) provided a good overview of the sea to the southeast with an observation height of approximately 47 m above sea level (m.a.s.l.). The harbour seal study was conducted at the northeastern tip of the Island of Anholt (Totten) in Kattegat, Denmark (figure 1), 1–20 September 2015. Totten, an area appointed as a seal reserve (closed to all public access), holds a significant seal population with approximately 1000 harbour seals and a few grey seals. The lighthouse located on the north coast enabled observations of the seal colony and the sea to the west of the colony with an observation height of approximately 40 m.a.s.l.

### 2.1. Simulating acoustic harassment devices sounds

An underwater loudspeaker (LL9162, Lubell Labs Inc., OH, USA) was used to transmit signals akin to that of the Lofitech AHD (Lofitech AS, Leknes, Norway), one of the most powerful and commonly used devices, but at a reduced peak-to-peak source level of 165 dB re 1 µPa (nominal source level of the Lofitech is 189 dB re 1 µPa root mean squared (RMS)). The lower source level was used to ensure that responses of seals and especially porpoises occurred within the range of the observers to allow reliable theodolite tracking. The signal frequency was chosen to be at 12 kHz, within the range of most commercial AHDs (10–14 kHz). Each signal was 0.5 s long and repeated with randomized intervals between 0.6 and 90 s, mimicking the workings of a Lofitech device. Signals were generated by a sound card (44.1 kHz) in a laptop computer by means of custom software developed in LabView, amplified by a 12 V car amplifier (Earth-quake 1000 W) and fed through a 300 m impedance matched cable to the loudspeaker. The loudspeaker was deployed 2 m above the seabed, suspended between a 25 kg concrete anchor and two trawl balls as flotation. Water depth in the study areas was 5–10 m for the porpoise study and 5–8 m for the seal study.

Transmitted signals were recorded by four stationary acoustic recorders (SoundTrap, model 202, Ocean Instruments, Auckland, New Zealand), deployed at distances of 104–310 m from the loudspeaker in the porpoise study and 86–205 m in the seal study (figure 1). Recorded signals were subsequently analysed in Matlab (v. 2014b). For each signal, the *L*_eq125 ms_ [21] was calculated as maximum value of a running RMS average with a 125 ms window on bandpass-filtered signals (1 kHz bandwidth centred around 12 kHz, fourth-order Butterworth), as well as the single pulse sound exposure level, SEL_SS_:

2.1 $$\text{SE}{\text{L}}_{\mathrm{SS}}=10\log_{10}\frac{1}{f_{s}}\sum p_{i}^{2},$$

where *p_i_* is the pressure measured in the *i*th sample and *f*_s_ the sample rate and summed over the duration of the signal. SEL_SS_ is related to the equivalent sound pressure level (RMS average) over the entire pulse duration (*d*) in a simple way:

2.2 $$L_{\mathrm{eq}}=\text{SE}{\text{L}}_{\mathrm{SS}}-10\log_{10}d.$$

For constant-amplitude signals *L*_eq_ over the entire signal and *L*_eq125 ms_ as calculated above will be identical, but as there is almost always some fluctuations in amplitude, the maximum *L*_eq125_ were up to a few decibels larger than *L*_eq_.

Positions of the loudspeaker and acoustic recorders were marked by orange surface buoys. Two additional dummy buoys without instruments were deployed at the outer corners of the study area (figure 1) to aid observers, when performing regular scans of the study area and to facilitate rapid communication of observations.

### 2.2. Localization and tracking of animals

Porpoises and seals were localized by a theodolite (Geodimeter 500, Geotronics AB, Sweden) communicating via a serial port (RS232) with the tracking software Cyclops Tracker (v. 2004, Eric Kniest, University of Newcastle, Australia), providing a real-time display of the localized animals. The theodolite was placed at the same location every day and the exact height of the station was determined daily. In the porpoise study, the height of a reference pole was measured by differential GPS by a certified surveyor. In the seal study, the known height of the lighthouse (height of light beam above sea level) was used. Variations in sea level owing to tide and weather were generally low, within approximately 1 m and were accounted for by measuring the vertical angle to the water surface on a fixed pole in the water. This was done approximately every hour and subsequent corrections for tide were performed by the Cyclops software.

Observation protocols differed between the two studies primarily owing to differences in the behaviour of harbour porpoises and harbour seals. Both studies were conducted whenever weather permitted, i.e. low winds and calm seas. As porpoises were more difficult to observe than seals owing to the fact that they often only surface very briefly and were in general further away from observers than seals, the weather criterion was set to sea state 2 or below for the porpoise study, while it was set to sea state 3 or below for the seal study. All animals were initially observed using naked eye and binoculars following a scanning protocol, which involved systematic scanning of the survey area (stretching from land and 100–200 m around the outer buoys) in three left–right bands. When an individual or group was sighted, it was localized using the theodolite.

In the porpoise study, three to four people were involved in the data collection at any time. Two observers were continuously scanning the study area with naked eye and binoculars to spot porpoises. Whenever one observer spotted one or more porpoises, the other observer manned the theodolite and started tracking the animal(s) by guidance of the first observer, while a third person checked the incoming position data in Cyclops and added comments to the experimental log. If a fourth observer was present, this person continued to scan the remaining area with binoculars. The tasks rotated among observers at regular intervals to prevent fatigue. In the seal study, the data collection involved four people. Two observers were continuously scanning the study area using naked eye and binoculars on each side of the theodolite. The theodolite was unmanned, so that both observers could use the device to localize animals, whenever they spotted a seal. A third person was stationed by a computer categorizing incoming observations and taking notes. Hence, three people were required for the observations and a fourth person allowed rotation between the posts every 20–30 min to stay focused.

### 2.3. Sound exposure trials

For porpoises, a trial with sound exposure was initiated whenever at least 1 h of baseline observations was made and a porpoise/a group of porpoises came within approximately 500 m of the loudspeaker. Sound exposures lasted for 15 min and observations were continued as long as possible, i.e. until the observers lost sight of the porpoise(s). Animals were often part of a group of two to four individuals. Groups were tracked as one, as it was not possible to identify individual animals within groups.

For seals, a trial included a minimum of 30 min baseline observation period, a 20 min sound exposure and a minimum of 30 min recovery period after each trial. Because seals can remain submerged for several minutes and a large number of seals were often scattered over a large area, individual seals could not be identified and hence not be tracked. An observation thus consisted of a surfacing of a single seal. Subsequent observations within a short time could thus both represent single observations of many seals or many, repeated observations of a few seals. At times with many seals visible at the same time, localization of the animals closest to the loudspeaker was prioritized.

A number of ‘blind’ trials were conducted in both experiments, four in the porpoise study and seven in the seal study. During these trials, the observers were unaware whether sound was played during the trial or not, but otherwise conducted as the remaining trials. These blind trials were included to assess possible observation bias caused by the observers' expectations of animal responses to sound.

### 2.4. Analysis

Repeated observations of the same group of porpoises were connected to form tracks. Ranges to the loudspeaker were calculated for each observation point together with swimming speed and direction inferred from surface observations. The response of each porpoise group was classified as avoiding the sound, if the range to the loudspeaker clearly increased during sound exposure and/or if animal swimming direction and speed changed drastically. In most cases, the number of observations during sound exposure was very low compared with the baseline period, preventing a proper analysis of the track, such as by application of algorithms intended to locate inflection points or similar discontinuities. However, a change in observation rate is in itself indicative of a change in behaviour of the porpoises, yet when too few observations were available, animals were classified as not responding. Everything else being equal, this will tend to underestimate the deterrence range of porpoises and is thus a conservative approach when the aim is to test for a difference from the expected smaller deterrence distance of seals.

Seal observations were grouped according to distance to the loudspeaker in bins of 50 m to assess changes in sighting rates. Generalized linear (mixed) models (GLMM; logarithmic link function) made in R (v. 3.2.2, R Development Core Team) were used to evaluate differences in observation counts within 100 m of the loudspeaker between sound and control trials, from baseline to exposure and from exposure to recovery. The explanatory variable was the observation count and included predictor variables were treatment (control/sound) and observation period (baseline, exposure and recovery) as factors, as well as their interaction term, with length of observation period used as a logarithmic offset to account for effort [22]. Different error distributions (Poisson) were tested to account for overdispersion (negative binomial) and zero inflation in the data using the glmmADMB package [23]. The optimal model fit based on lowest Akaike's information criteria (AIC) included a negative binomial distribution and no zero inflation. To test for development of responses over time (possible habituation or sensitization to sound with multiple exposures), trial number as a continuous variable (with controls set to zero) was initially also assessed as predictor variable. However, a better model fit was obtained when trial number was included as a random factor. Inclusion of a random effect allows the model to account for variation in the dataset, but is not of primary interest in the study, and in this context, also allow observations within each trial to be correlated [22].

## 3. Results

### 3.1. Sound exposure

**Figure 2.**
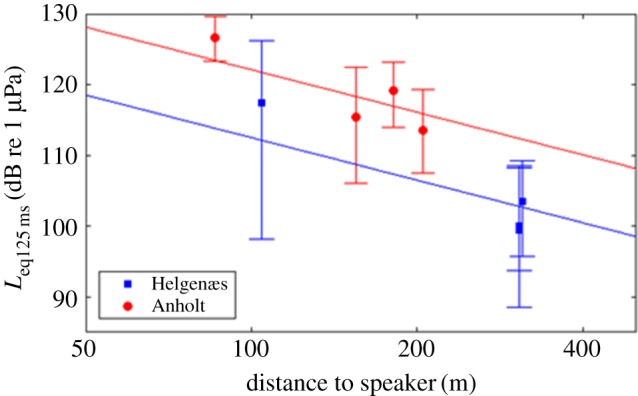
Sound pressure levels with range from loudspeaker. The median sound pressure level over a 125 ms window (*L*_eq125 ms_) with error bars indicating 95th and 5th percentiles at each of the four stations at both study areas. The two lines are best fitting lines with a slope of 20** **dB per decade, corresponding to spherical spreading loss without absorption. Corresponding source levels (*L*_eq125 ms_) back calculated to 1** **m were 152.5** **dB re 1 µPa and 162.1** **dB re 1 µPa for the porpoise study area and the seal study area, respectively.

**Figure 3.**
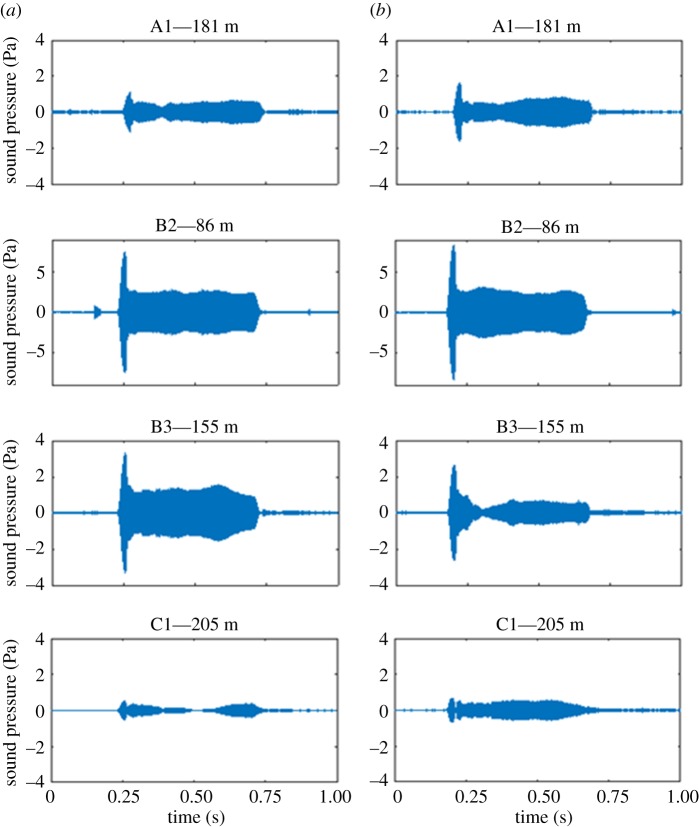
Examples of two consecutive AHD signals (*a*,*b*). The two signals are separated by about 40 s and recorded simultaneously on all four stations at the seal study area (Anholt) (see positions in figure 1). Note that the *y*-axis at station B2 differs from the rest.

Simulated AHD sounds recorded at the four acoustic recording stations were evaluated to provide estimates of the noise exposure levels that porpoises and seals experienced (table 1). Recordings showed a decrease in sound pressure level with range to the loudspeaker as expected (figure 2). However, results also showed a very large variation in sound pressure level, 20 dB or more (figure 2). This variation was both within trials (i.e. from pulse to pulse, as illustrated in figure 3) and between trials and was evident both in sound pressure levels (*L*_eq125 ms_) and sound exposure levels (SEL_SS_, table 1). Power density spectra (Welch averages) showed that harmonics were more than 40 dB lower than the main peak at 12 kHz and thus unlikely to contribute to the overall audibility of the signal.

**Table 1.** Sound pressure level (*L*_eq125 ms_) and sound exposure level (SEL_SS_) of seal scarer pulses in the two study areas. (Values are shown for the 50th (median), 95th and 5th percentiles. *L*_5_ is thus the level exceeded by 5% of the pulses.)

	range	*L*_50_	*L*_95_	*L*_5_	SEL_50_	SEL_95_	SEL_5_
station	m	dB re 1 µPa			dB re 1 µPa^2^s		
porpoise study (Helgenæs)							
A1	307	99.4	88.5	108.4	94.0	83.4	103.2
B2	104	117.5	98.2	126.2	112.0	92.7	121.2
B3	310	103.5	95.7	109.2	97.9	90.1	104.4
C1	307	100.0	93.7	108.2	94.5	88.3	103.9
seal study (Anholt)							
A1	205	113.6	107.5	119.3	108.2	102.2	114.4
B2	86	126.6	123.3	129.7	121.1	117.8	124.0
B3	181	119.1	113.9	123.1	113.7	108.1	117.8
C1	155	115.4	106.1	122.4	109.6	100.4	116.5

### 3.2. Responses of porpoises

Sixteen trials were conducted over 9 days in June–July 2015, when weather conditions permitted tracking of porpoises (sea state 0–2, wind speed less than 6 m s^−1^). Of these, 12 trials were with sound and four were ‘blind’ control exposures (table 2). Tracked porpoises were either individuals or groups (i.e. only one individual/group was tracked per trial, except in one case where two distinctively separated groups could be followed at the same time). In three cases, porpoises were lost out of sight shortly after the initiation of sound exposure. These animals were classified as not evading the sound, although they appeared to have responded and probably evaded the sound (marked in table 2), leading to an underestimation of deterrence range. In 10 trials, porpoises were observed multiple times after sound exposures and their responses could therefore be properly evaluated. In seven of these cases, porpoises were classified to have reacted to the simulated AHD sounds by avoidance (table 2).

**Table 2.** Information about sound exposure trials for harbour porpoises. (Included is the range between the loudspeaker and the porpoise(s) at the last observation before sound is turned on (distance before), the minimum distance observed after exposure start, and the minimum observation after 5 min of exposure (or the last observation made if the animal disappeared, marked by parentheses). If the animals were judged to respond by avoidance, they were assigned a ‘Yes’ in the far right column (avoidance). The first four trials were conducted as blind controls, where the observers were unaware of whether sound was played or not (indicated by ‘No’ in the ‘sound’ column). Tracklines for all exposures can be seen in the electronic supplementary material, S1 and S2.)

trial no.	date	time	no. animals	distance before	min. distance	min. distance after 5 min of exposure	sound	avoidance
1	3 July 2015	10.12.33	2–3	457	450	512	No	—
2	3 July 2015	12.37.36	2	539	425	425	No	—
3	3 July 2015	16.19.02	2	340	370	385	No	—
4	4 July 2015	10.49.29	2	407	324	386	No	—
5	4 July 2015	15.34.12	1	189	138	310	Yes	Yes
6	4 July 2015	18.01.46	1	525	525	602	Yes	Yes
7	5 July 2015	07.40.58	2	396	193	540	Yes	Yes
8	5 July 2015	10.13.29	2	435	401	910	Yes	Yes
9a	11 July 2015	09.36.40	2	479	412	790	Yes	Yes
9b	11 July 2015	09.36.40	2	422	571	498	Yes	No
10	12 July 2015	10.10.29	2	553	553	(607)	Yes	No^a^
11	12 July 2015	11.52.31	1–2	645	546	(546)	Yes	No^a^
12	12 July 2015	13.08.48	2–4	659	645	716	Yes	No^a^
13	12 July 2015	14.41.27	2	351	351	475	Yes	No
14	12 July 2015	16.00.07	2	421	354	486	Yes	No
15	13 July 2015	09.23.32	1–2	343	343	423	Yes	Yes
16	14 July 2015	11.31.31	2	357	157	301	Yes	Yes

^a^The animals in these trials disappeared just after sound was initiated and could not be tracked further, obstructing evaluation of a response. As a precautionary approach, they were set as ‘No’ response.

**Figure 4.**
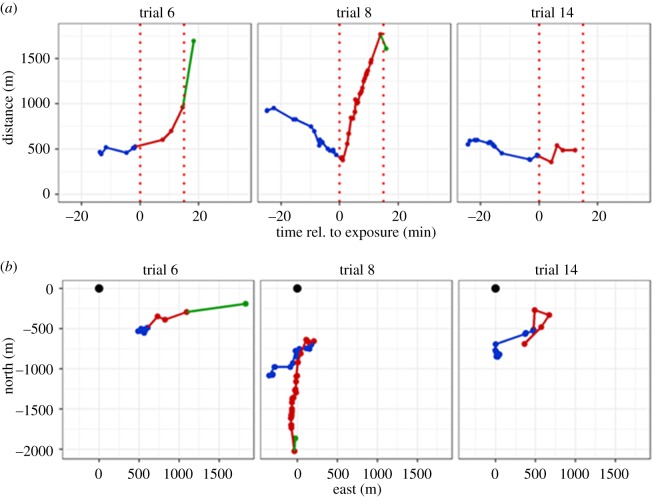
Examples of track lines of harbour porpoises. Baseline observations (blue), observations made during sound exposure (red), and observations during recovery (green) are shown. (*a*) Observations plotted as distance to the loudspeaker relative to the sound exposure at time zero. Red stippled lines indicate the period of sound exposure. (*b*) The same track lines displayed as geographical coordinates relative to the position of the loudspeaker (black dot) at position (0, 0).

**Figure 5.**
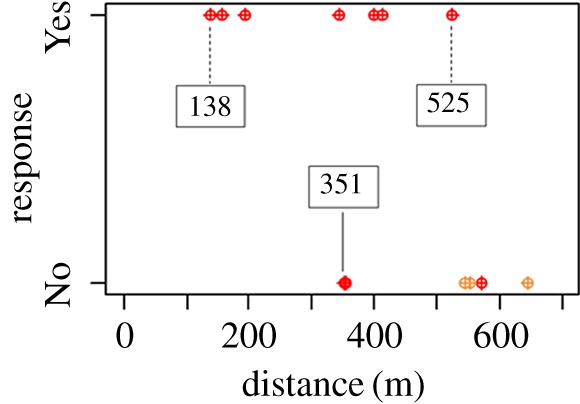
Evaluated avoidance responses (‘Yes’ or ‘No’) of harbour porpoises for all sound exposures plotted with the estimated minimum distance to the loudspeaker, when a response was observed (red dots). The dots in orange colour indicate the three unclear responses (table 2), evaluated as ‘No’ response as a conservative approach.

Three examples of tracked porpoise groups during sound exposure trials are shown in figure 4. In the first example (trial 6), a single animal at an initial distance of approximately 500 m from the loudspeaker reacted with avoidance and moved to a range of approximately 1700 m within 18 min, although it did not move in a straight path away from the loudspeaker in the first half of the exposure. In the second example (trial 8), two animals were approximately 300 m from the loudspeaker when the sound was turned on, and responded strongly by avoidance on a path directed away from the loudspeaker out to a range of approximately 1700 m within 15 min. Animals in both trials (trials 2 and 4) were evaluated to have responded to sound (‘Yes’ response, table 2) based on their tracks before, during and after the sound exposure. By contrast, the third example (trial 14) shows two animals, which were judged not to have reacted to the sound exposure (‘No’ response, table 2), as this group did not swim away during playback of the sound, although they were initially at approximately 350 m from the loudspeaker, but instead stayed within 500 m of the loudspeaker throughout the trial. Table 2 summarizes information from all exposures including an evaluation of whether there was a response to the simulated AHD sound or not. All individual tracks can be found in the electronic supplementary material, S1 and S2. Figure 5 summarizes all the responses based on a ‘Yes’ or ‘No’ criteria to a flight response. All animals within approximately 190 m (trials 5, 7 and 16, table 2) to the simulated AHD sounds, and animals out to a distance of 525 m were judged to exhibit an avoidance response (figure 5 and table 2).

### 3.3. Responses of seals

**Figure 6.**
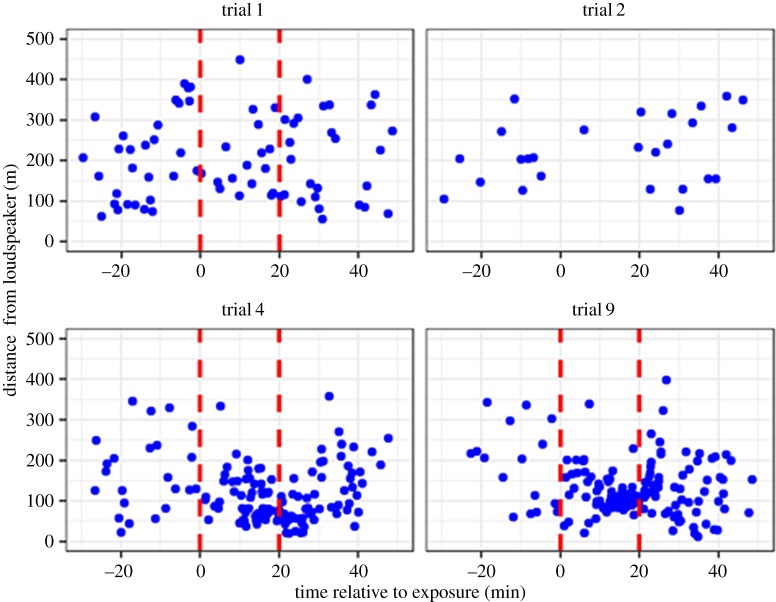
Examples of seal observations plotted as distances from loudspeaker (m) relative to time of exposure (min). The red stippled lines indicate onset and end of sound exposure. Three sound exposure trials (trials 1, 4 and 9) and one control trial (trial 2) are shown.

**Figure 7.**
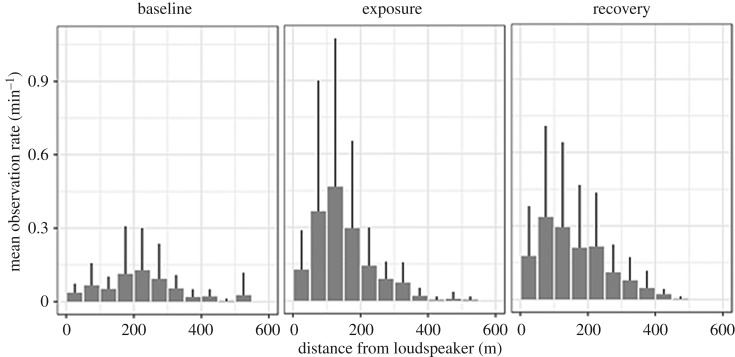
All ranges of harbour seals relative to the loudspeaker observed during 13 sound exposure trials displayed as the mean number of seal observations per 50 m bins within the three time periods, baseline, sound exposure and recovery. Positive standard deviations are shown.

**Figure 8.**
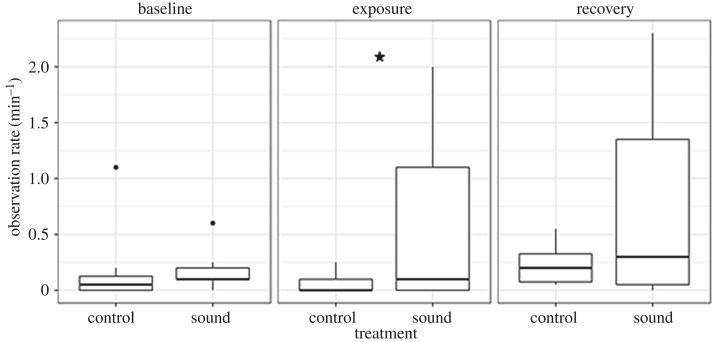
Frequency of seal observations (per minute) within 100 m of the sound source during control and sound trials in the three treatment periods; baseline, exposure and recovery. The asterisk depicts significance.

A total of 20 trials were conducted during seven fair weather days in September 2016 with 13 trials exposing seals to sound and seven trials being controls (figure 6). The number of seal observations differed markedly between trials. Figure 6 compares observations in four trials: during trials 4 and 9, a change in the distribution of seals could be seen during sound exposure, with seals moving closer to the loudspeaker (within approx. 10 m in trial 9). This pattern was not evident in trial 1, where observations were distributed more evenly during all periods, similar to observations in trial 2 without sound (control) (all trials can be found in the electronic supplementary material, S3). Across all sound exposure trials, however, seals were in general observed more often and closer to the loudspeaker during the exposure and recovery period, in comparison to the baseline (figure 7). The highest observation rate per minute during baseline was in the distance interval 201–250 m from the loudspeaker, in comparison to seals being observed more often at closer ranges of 101–150 m during exposure and 51–100 m during recovery (figure 7). The increasing number of seal observations close to the loudspeaker during (and after) sound exposure can also be seen in figure 8, illustrating that seals were observed more often during sound trials than control trials within 100 m of the loudspeaker. This was supported by the GLMM, which showed that the interaction term between the treatment, (sound), and the observation period, (exposure), was marginally significant (*p* = 0.044). The model coefficient for this interaction indicated a 4.5 times increase in seal observations in the exposure period during sound treatment compared with control, however with large variation between trials as indicated in figure 8 (all model terms can be found in the electronic supplementary material, S4). There was no clear sign of habituation to the AHD sounds in seals, as trial number as a continuous variable was not to prefer over a model with trial number as random factor (AIC 345 and 329, respectively).

## 4. Discussion

Here, we demonstrate a large difference in responsiveness to the same AHD-type signal in two different, but sympatric marine mammals. Harbour porpoises reacted by avoidance at distances of several hundred metres from the loudspeaker, whereas harbour seals remained around the loudspeaker and were even observed to approach it during playbacks.

The majority of porpoises within 525 m of the loudspeaker moved away from the sound source when the AHD sound was on. This distance corresponds to an estimated median received sound pressure level (*L*_eq125 ms_) of 98 dB re 1 µPa (based on the source level estimate from figure 2 and a spherical spreading loss model). All porpoises within 190 m from the source reacted with avoidance (figure 5) but beyond approximately 350 m, the reaction was not consistent, as an avoidance response was not observed with certainty in all animals. A single animal approached the loudspeaker to a distance of only 157 m before leaving the area (trial 12, table 2). The estimated sound pressure level (*L*_eq125 ms_) at 190 m from the loudspeaker was 107 dB re 1 µPa. This threshold is in agreement with other studies, where wild porpoises were exposed to a Lofitech AHD. Brandt *et al.* [18] found that porpoise acoustic activity (as assessed by passive acoustic monitoring) decreased out to a distance of 7.5 km from a Lofitech AHD, where the received sound pressure level (*L*_eq_ over the pulse duration) was estimated to be 113 dB re 1 µPa at the reaction. Yet, a few porpoise clicks were detected at 750 m, which would mean that some animals tolerated a considerably higher sound pressure level (RMS average, *L*_eq_, over the pulse duration) of approximately 139 dB re 1 µPa. Brandt *et al.* [19] later conducted a visual theodolite study similar to the study conducted here, where all observed porpoises were found to avoid the Lofitech AHD within 1.9 km of the transducer, corresponding to estimated *L*_eq_ ≥ 122 dB re 1 µPa, and half of the animals at 2–2.4 km range exhibited an observable avoidance response (estimated *L*_eq_ of 119 dB re 1 µPa). The closest approach was found to be around 800 m (estimated *L*_eq_ 132 dB re 1 µPa). In a few other studies conducted on wild porpoises, the Airmar dB plus II AHD was tested and the minimum deterrence distance was estimated to be 200 [9] and 645 m [8] corresponding to porpoises avoiding areas where exposure levels of 148 (based on 194 dB re 1 pPa at 1 m (peak-to-peak) and spherical spreading loss) and 128 dB re 1 µPa_pp_, respectively. The differences among the various studies and between the two different AHDs could be related to the different signals of the Airmar and the Lofitech signals [7], also noting differences in units (peak-to-peak for the Airmar, versus *L*_eq_ for the Lofitech), differences in the sound propagation properties or observation methods, and probably also a context dependency. Animals engaged in important activities, such as foraging, are in general less likely to respond to disturbing sounds compared with animals that are merely travelling from one place to another [16,24,25]. Overall, however, there is good agreement between the present results (response threshold, when expressed as *L*_eq125 ms_, in the range 98–107 dB re 1 µPa and the observations of Tougaard *et al.* [21], which predicts response thresholds based on the sensation level of the signals: around 45 dB above the pure tone hearing threshold, or equal to about 100 dB re 1 µPa at 12 kHz.

The harbour seals, in contrast with the porpoises, did not avoid the sound source at all. Instead, they were observed more often and closer to the loudspeaker during sound exposures than in the preceding control periods. This could indicate that the sound elicited curiosity rather than fear in the seals. It was therefore not possible to estimate deterrence thresholds for the seals owing to their lack of an avoidance response to the received levels generated in this study. It could be argued that seals were observed more often simply because they lifted their heads out of the water more often to avoid the noise [26,27]. However, the distribution of seals changed from baseline to exposure (figure 5) and seals were also observed to approach the study site during exposure. Some seals were observed to be within approximately 10 m of the loudspeaker during sound exposures, corresponding to a received level (median *L*_eq125 ms_) of at least 142 dB re 1 µPa (based on the source level estimate from figure 2 and spherical spreading loss). The lack of seal deterrence observed here is, however, in line with results from the few other studies, where effects of AHD have been tested on wild seals in contexts not related to fishery or foraging sites. Gordon *et al.* [17] found that all seals they observed out to a distance of approximately 1000 m from a Lofitech AHD responded to the playback. However, not all animals reacted by moving directly away from the sound source and some individuals were often seen at closer ranges. The absolute deterrence range of all animals was therefore estimated by Gordon *et al.* [17] to be 225 m with a predicted received sound pressure level (*L*_eq_) of 145 dB re 1 µPa. Graham *et al.* [28] tested the effectiveness of the Lofitech for excluding seals from Atlantic salmon rivers in Scotland in 2006 and found closest approaches of seals to be within 200 m of the AHD. Jacobs & Terhune [15] found no significant reactions of harbour seals to an Airmar dB II plus device with a source level of 178 dB re 1 µPa. They found that most seals in an area where AHDs were commonly used stayed approximately 200 m from the device, but a few individuals were observed as close as approximately 45 m. Götz & Janik [16] tested reactions of grey seals to sounds from different AHDs, including Lofitech, Ace-Aquatec and Airmar dB II plus devices. They found deterrence ranges of 40–60 m for grey seals, with least effects of the Airmar dB II plus device, corresponding to received sound pressure levels (*L*_eq_) at threshold between 135 and 144 dB re 1 µPa. Because of the different methods and sound types/levels used in these studies, it is therefore difficult to compare the results directly and draw firm conclusions on response thresholds. Another factor that may explain differences in responses is the context of the study site with respect to environmental properties, such as distance to land and water depth relating to an animal's ability to escape a potential danger. This study was conducted close to a haul out site, as in Jacobs & Terhune [15] and Götz & Janik [16], where seals may react differently than in more open water, as in the study by Gordon *et al*. [17]. Habituation may also influence the responsiveness. If seals are used to exposure to AHDs [7], or if they link AHD sounds with fish (dinner bell effect) [29], their response thresholds are likely to be higher than for naive seals such as in this study. Harris *et al.* [30] found that some seals (mainly a few grey seals) came within 80 m of a Lofitech AHD, when testing its effectiveness of keeping seals away from Atlantic salmon net fishery. As this study was conducted in a real fishery, a strong food incentive may have caused the seals to tolerate a higher source level. Also, these close sightings only occurred during the second year of the study, indicating habituation or increased tolerance over time. However, no clear indication of habituation was found in our data, and because AHDs are not commonly used in Denmark in fishery-related contexts, an attraction because of a conditioned dinner bell effect is unlikely.

Variation between the different studies and the large variation observed in responsiveness of individuals in our study can probably also be explained to some degree by the fact that received sound levels fluctuated considerably from signal to signal (figures 2 and 3) as also shown previously by Shapiro *et al.* [31]. This variability is most likely related to the relatively long pure tone pulses (0.5 s) combined with the shallow water environment. Multi-path propagation and reflections from surface and sea floor will form patterns of constructive and destructive interference in the signals, in particular for long pure tones. This interference most likely caused the pronounced initial peak in the signals (figure 3), as this is the only part of the directly transmitted signal not interfered with by the surface reflection. Variation could be further increased from trial to trial owing to differences in sea state (even small changes to the roughness of the sea surface will have large effects on reflections) and differences in suspended materials (large amounts of suspended silt and clay following the hours and days after heavy winds will increase scattering of the sound considerably). However, the overall good correspondence with spherical spreading out to the maximum distance measured (300 m) is in line with previous studies on the same type of signal in shallow water [31] and very likely reflects the large variability in received levels from one pulse to the next that will be experienced by an animal swimming around a real ADH.

In conclusion, this study presents data showing that harbour porpoises exhibit much stronger avoidance responses to simulated AHD sounds than harbour seals do. While all porpoises avoided the sound source when exposure levels exceeded 107 dB re 1 µPa (median *L*_eq125 ms_), corresponding to about 50 dB sensation level above the pure tone hearing threshold, and some porpoises also reacting to levels down to 98 dB re1 µPa (median *L*_eq125 ms_), seals instead did not evade the sound source and some individuals were observed approaching the active sound source at received sound levels exceeding 142 dB re 1 µPa (*L*_eq125 ms_), i.e. a difference of more than 40 dB.

This difference of at least 40 dB in reaction thresholds has large implications for the deterrence ranges and the associated temporary habitat loss caused by the ADH. In a thought example, take a 12 kHz signal, as the one used in our experiments, adjusted in amplitude so to obtain a deterrence range of, say, 100 m for harbour seals, the corresponding deterrence range for harbour porpoises under simple assumptions of spherical spreading and absorption of 1 dB km^−1^ will be about 5 km, as the received level at this range will be 40 dB below the level at 100 m. The difference in scale of the habitat area is dramatic: 0.03 km^2^ for the seals; 80 km^2^ for the porpoises. The inescapable conclusion is thus, that to use an AHD such as the Lofitech or similar device with a signal equally audible to seals and porpoises to deter seals from pile driving noise or similar, may constitute a disproportionally large habitat loss to harbour porpoises in the same area. This problem is exacerbated even more if the AHD is used to deter seals from aquaculture or fishing gear, as the seals can be expected to tolerate even higher noise levels, owing to the positive reinforcement caused by the food reward offered by the net pen or fishing gear [32].

A solution to this problem, however, offers itself readily and this is exploiting the differences in hearing sensitivity between seals and porpoises for frequencies further below 12 kHz as proposed and tested by Götz & Janik [20,33]. Thus, development of devices with signals more audible to the target species could limit the side effect of scaring harbour porpoises and other species unnecessary far away from areas with fishery and aquaculture installations where AHDs are regularly used. For mitigation purposes, it could provide a more balanced deterrence, and avoid the current situation where the mitigation itself (the AHD) for porpoises could present an impact in the form of temporary habitat loss equal to or even exceeding the very impact it should mitigate.

## Supplementary Material

S1. Track lines of harbour porpoises as distances from loudspeaker

## Supplementary Material

S2. Track lines of harbour porpoises as geographical coordinates

## Supplementary Material

S3. Observations of harbour seals

## Supplementary Material

S4. GLMM model coefficients and confidence intervals.

## Data accessibility

Data are available from the Dryad Digital Repository: (http://dx.doi.org/10.5061/dryad.8jh04) [34].
