# Supplementary figures and images for: Simulated seal scarer sounds scare porpoises, but not seals: species-specific responses to 12 kHz deterrence sounds
Source: R Soc Open Sci. 2017 Jul 19;4(7):170286. doi: 10.1098/rsos.170286 (PMC5541550; doi:10.1098/rsos.170286)

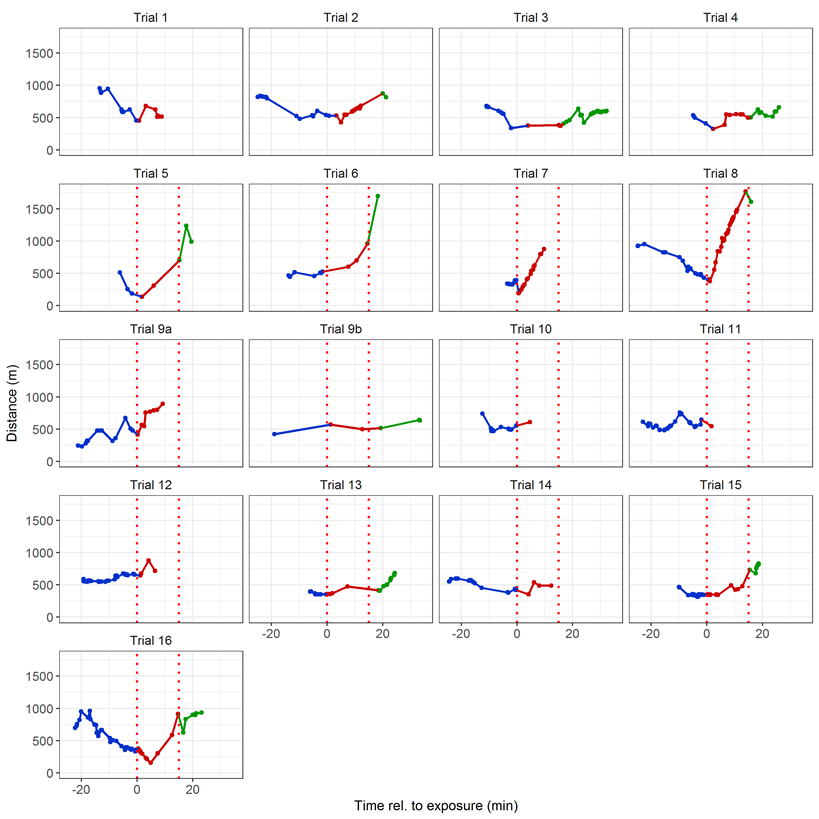

Supplement: S1. Track lines of harbour porpoises as distances from loudspeaker [file rsos170286supp1.tif]

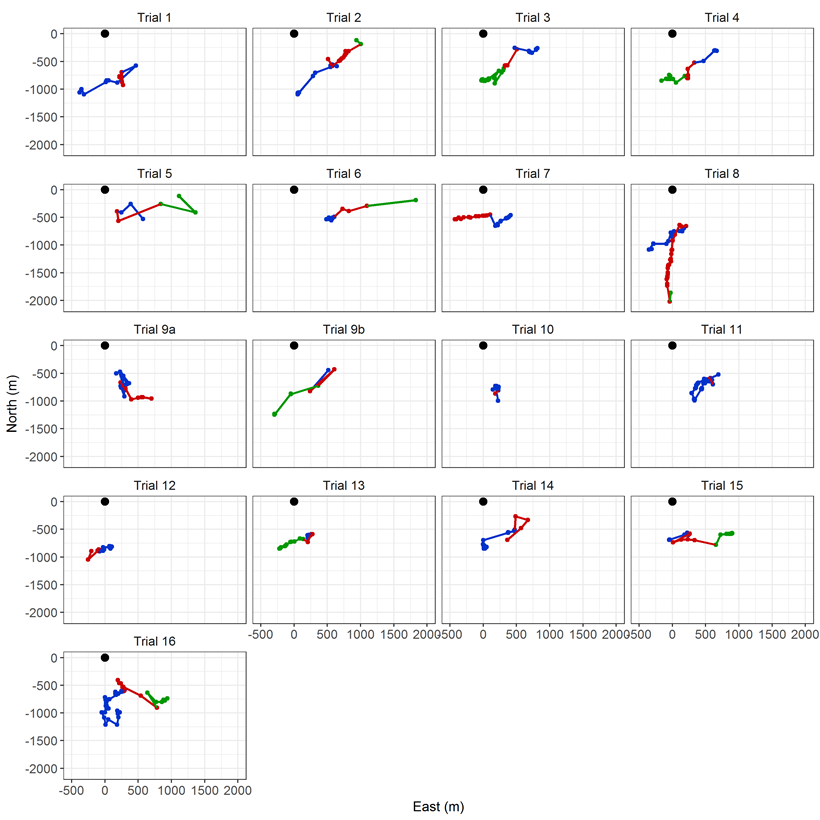

Supplement: S2. Track lines of harbour porpoises as geographical coordinates [file rsos170286supp2.tif]

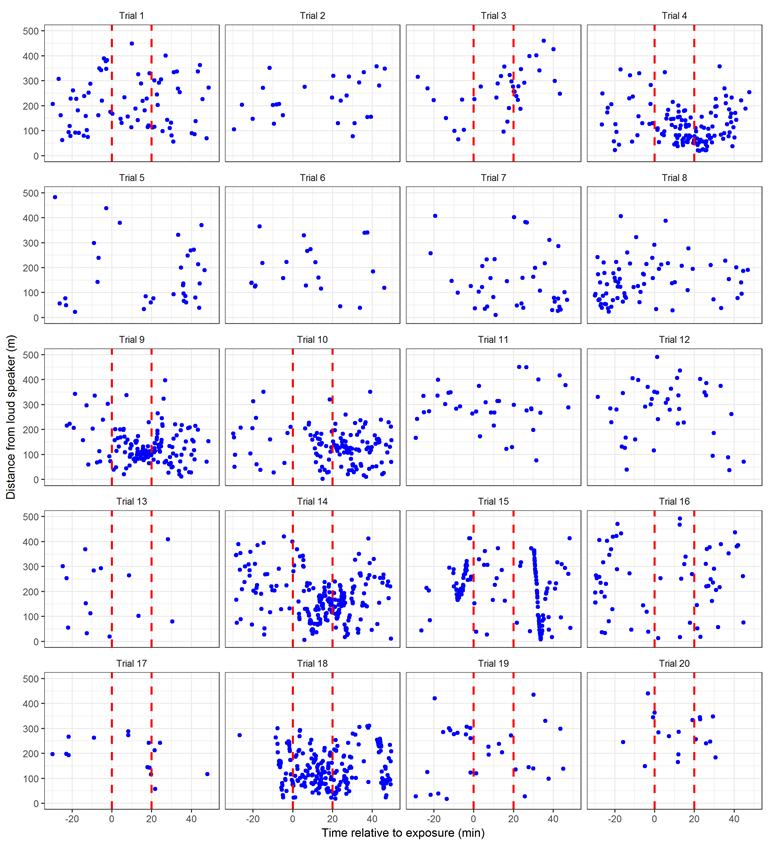

Supplement: S3. Observations of harbour seals [file rsos170286supp3.tif]
