# Supplementary material for: Simulated seal scarer sounds scare porpoises, but not seals: species-specific responses to 12 kHz deterrence sounds
Source: R Soc Open Sci. 2017 Jul 19;4(7):170286. doi: 10.1098/rsos.170286 (PMC5541550; doi:10.1098/rsos.170286)
Supplement: S4. GLMM model coefficients and confidence intervals. [file rsos170286supp4.docx]

S4. Model coefficients and confidence intervals from the Generalised Linear Mixed Model (GLMM) applied on the seal count data with a negative binomial distribution. Trial number is included as a random effect, and treatment (sound/control) and observation period (baseline, exposure, recovery) are included as the predictor variables.

| **Predictor** | **Coefficient**  **e^β^** | **Confidence interval** | | **p** |
| --- | --- | --- | --- | --- |
|  |  | **2.5 %** | **97.5 %** |  |
| Intercept | 0.051 | 0.014 | 0.191 | < 0.001 |
| Sound | 1.231 | 0.240 | 6.301 | 0.803 |
| Exposure | 0.670 | 0.193 | 2.334 | 0.530 |
| Recovery | 2.171 | 0.716 | 6.572 | 0.170 |
| Sound * Exposure | 4.531 | 1.042 | 19.721 | 0.044 |
| Sound * Recovery | 1.815 | 0.470 | 6.998 | 0.387 |
